# Correction: Zhang et al. Construction of a Prognostic and Early Diagnosis Model for LUAD Based on Necroptosis Gene Signature and Exploration of Immunotherapy Potential. *Cancers* 2022, *14*, 5153

**DOI:** 10.3390/cancers18111696

**Published:** 2026-05-22

**Authors:** Baizhuo Zhang, Yudong Wang, Xiaozhu Zhou, Zhen Zhang, Haoyu Ju, Xiaoqi Diao, Jiaoqi Wu, Jing Zhang

**Affiliations:** 1 Department of Pharmacology, College of Pharmacy, China Medical University, Shenyang 110122, China; 2020120548@stu.cmu.edu.cn (B.Z.); 2020120590@stu.cmu.edu.cn (X.Z.); 2019110055@stu.cmu.edu.cn (Z.Z.); 2021120523@cmu.edu.cn (H.J.); 2021120538@cmu.edu.cn (X.D.); 2020120568@stu.cmu.edu.cn (J.W.); 2 Thoracic Surgery Department, Shengjing Hospital of China Medical University, Shenyang 110004, China; yudongwang@cmu.edu.cn

**Error in Figures**

In the original publication [1], there was a mistake in Figure 8D and the fifth panel of Figure 12C as published. Figure 8D was duplicated with Figure 8A, and the fifth panel of Figure 12C was duplicated with the first panel. The corrected Figure 8 and Figure 12 appear below. The authors state that the scientific conclusions are unaffected. This correction was approved by the Academic Editor. The original publication has also been updated.

## Figures and Tables

**Figure 8:**
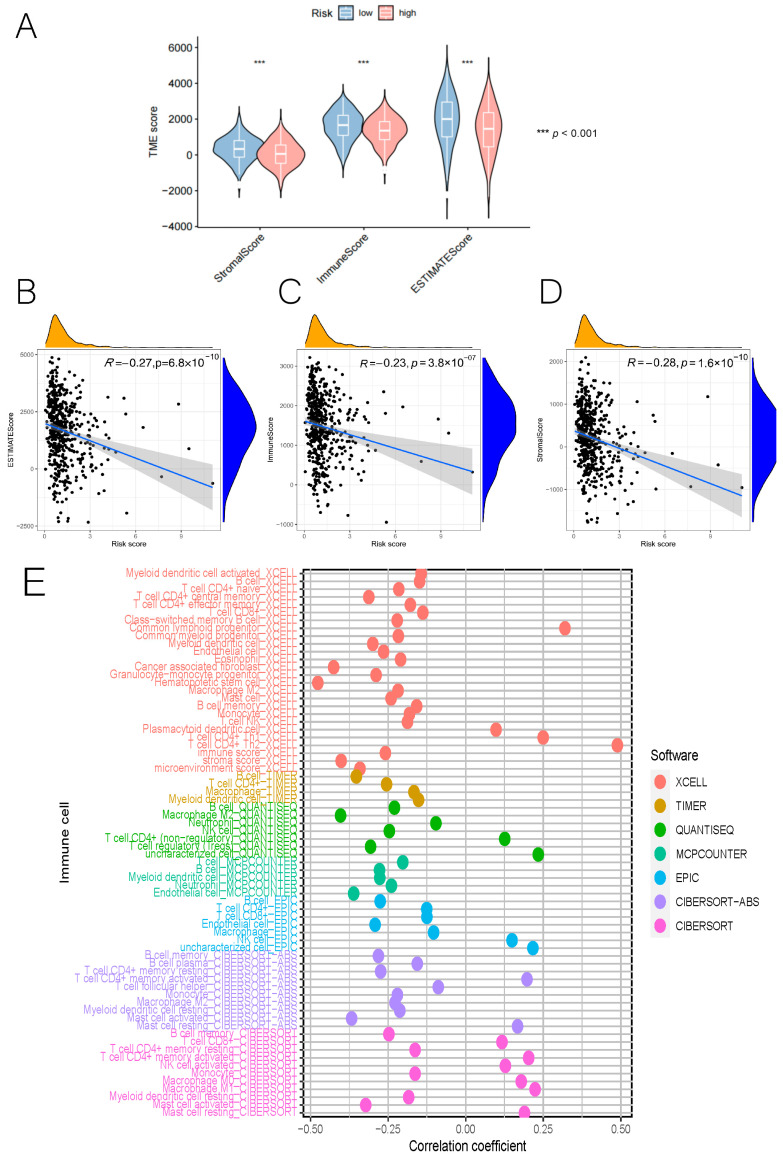
(**A**) Relationship between risk score and tumor microenvironment. (**B**–**D**) Correlation of StromalScore, ImmuneScore, and ESTIMATEScore with risk score. (**E**) Analysis of risk score correlation with immune cell infiltration based on 7 algorithms. *** *p* < 0.001.

**Figure 12:**
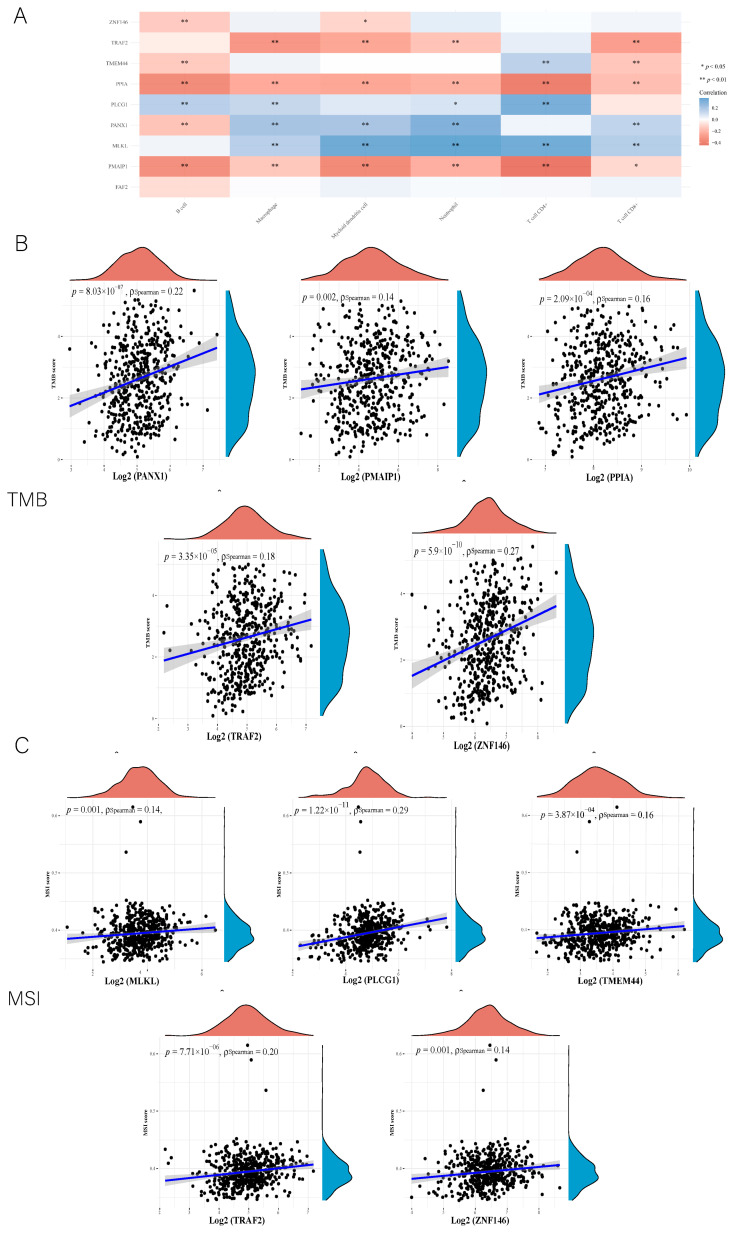
(**A**) Correlation of DENRGs with immune cells in the prognostic model. (**B**) DENRGs associated with TMB in the prognostic model. (**C**) DENRGs associated with MSI in the prognostic model. * *p* < 0.05 and ** *p* < 0.01.
